# Investigating the gut microbiome in schizophrenia cases versus controls: South Africa’s version

**DOI:** 10.1007/s10048-025-00816-9

**Published:** 2025-03-05

**Authors:** Carlien Rust, Laila Asmal, Michaela O’Hare, Etheresia Pretorius, Robin Emsley, Soraya Seedat, Sian Hemmings

**Affiliations:** 1 Department of Psychiatry, Faculty of Medicine and Health Sciences, Stellenbosch University, Cape Town, South Africa; 2 South African Medical Research Council / Stellenbosch University Genomics of Brain Disorders Research Unit, Stellenbosch University, Cape Town, South Africa; 3 Department of Biomedical Sciences, Division of Molecular Biology and Human Genetics, Faculty of Medicine & Health Sciences, Stellenbosch University, Cape Town, South Africa; 4 Department of Physiological Sciences, Faculty of Science, Stellenbosch University, Stellenbosch, South Africa; 5 Department of Biochemistry and Systems Biology, Institute of Systems, Molecular and Integrative Biology Biosciences Building, University of Liverpool, Liverpool, UK

**Keywords:** Schizophrenia, Gut microbiome, Gut-brain-axis, Alpha-diversity, Beta-diversity

## Abstract

Schizophrenia (SCZ) is a chronic and severe mental disorder with a complex molecular aetiology. Emerging evidence indicates a potential association between the gut microbiome and the development of SCZ. Considering the under-representation of African populations in SCZ research, this study aimed to explore the association between the gut microbiome and SCZ within a South African cohort. Gut microbial DNA was obtained from 89 participants (*n* = 41 SCZ cases; *n* = 48 controls) and underwent 16S rRNA (V4) sequencing. Data preparation and taxa classification were performed with the *DADA2* pipeline in R studio followed by diversity analysis using *QIIME2*. Analysis of Compositions of Microbiomes with Bias Correction (ANCOM-BC) was utilised to identify differentially abundant taxa. No statistically significant differences were observed between SCZ patients and controls in terms of alpha-diversity (Shannon *q* = 0.09; Simpson *q* = 0.174) or beta-diversity (*p* = 0.547). Five taxa, namely *Prevotella* (*p* = 0.037)*, Faecalibacterium* (*p* = 0.032)*, Phascolarctobacterium* (*p* = 0.002)*, Dialister* (*p* = 0.043)*,* and *SMB53* (*p* = 0.012), were differentially abundant in cases compared to controls, but this observation did not survive correction for multiple testing. This exploratory study suggests a potential association between the relative abundance of *Prevotella, Faecalibacterium, Phascolarctobacterium, Dialister,* and *SMB53* with SCZ case–control status. Given the lack of significance after correcting for multiple testing, these results should be interpreted with caution. Mechanistic studies in larger samples are warranted to confirm these findings and better understand the association between the gut microbiome and SCZ.

## Introduction

Schizophrenia (SCZ) is a complex, multifactorial psychiatric disorder with an estimated prevalence of 0.5–1% in South Africa [1, 2]. To date, no single cause for SCZ has been established [3, 4] as both genetics [5] and the environment contribute to the disorder’s aetiology [6]. One way to assess the possible association between host genes and the environment could be through the gut microbiome as alterations in the gut microbiome are influenced by both genetics [5, 7] and environmental factors such as diet, medication, age, gender, lifestyle, geography, and population demographics [8, 9].

The gut microbiome plays a vital role in maintaining intestinal barrier integrity, modulating the immune system, and communicating with the central nervous system (CNS)[10]. Communication with the CNS is bidirectional and occurs via the microbiota-gut-brain (MGB) axis through the involvement of the vagus nerve and bacterial metabolites such as neurotransmitters, short-chain fatty acids (SCFA), and lipopolysaccharides (LPS) [11–13]. Alterations in the diversity and composition of the gut microbiome could influence immune responses [11] and may potentially lead to chronic systemic inflammation through altered levels of bacterial products including, but not limited to, LPS and SCFA [5, 10, 14]. Increased inflammation and disruptions in SCFA production by commensal bacteria, which could further exacerbate inflammation by compromising gut barrier integrity, have previously been associated with SCZ [4, 15]. A compromised gut barrier integrity could facilitate the translocation of bacterial metabolites across the gut epithelial barrier to promote chronic low-grade inflammation in individuals with SCZ by stimulating the expression of proinflammatory cytokines [9, 10, 14].

Differences in the diversity and composition of the gut microbiome and production of bacterial metabolites can indirectly affect brain networks, neurodevelopment, neuroendocrine and neurotransmission processes that contribute to SCZ aetiology [5, 15]. Further, mediation analyses have shown structural and functional brain networks to be associated with the gut microbiome in SCZ cases [16]. The increased translocation of LPS from the gut to the blood [17] and the decreased SCFA concentrations may affect the permeability of the blood–brain barrier (BBB) [9]. A compromised BBB enables the passage of pathogens and proinflammatory mediators, including LPS, into the brain [9]. This can activate microglial cells to release nitrogen and oxygen species that could further compromise the integrity of the BBB [18, 19]. In addition, overactivation of microglia could result in inflammation in the brain, often referred to as neuroinflammation [20]. Increased neuroinflammation has been associated with impaired social and cognitive behaviour, both of which are known to be symptoms of SCZ [21].

Several 16S rRNA studies have reported gut microbial differences between individuals with and without SCZ [3, 4, 9, 10, 14, 22]. Whilst some [23], but not all [22, 24–28], studies have observed alterations in alpha-diversity of the gut microbiome in individuals with SCZ, differences in beta-diversity are more consistently reported between individuals with SCZ and controls [3, 4, 10, 14, 22, 29, 30]. Despite this, it has proved difficult to identify a single microbial profile associated with the disorder [31]. Previous reports have highlighted the possible involvement of *Prevotella* [3], *Collinsella* [32]*, Roseburia* [26], *Faecalibaterium* [10, 14], *Clostridium* [4], *Lactobacillus* [10], *Ruminococcus* [4, 14, 26], and *Haemophilus* [32]. In general, alterations in these gut bacteria point to the involvement of SCFA production, immune response modulation, and intestinal barrier permeability potentially playing a role in SCZ. Additionally, faecal microbial transplants from SCZ patients to germ-free mice have demonstrated the importance of the gut microbiome in disorder, as altered microbial communities could contribute to altered neurotransmitter levels and SCZ-like behaviours [26, 32, 33].

To date, no reported studies have investigated the gut microbiome and SCZ in South Africa. Our study sought to investigate whether the gut microbial profile of individuals with SCZ differed from those without SCZ within a South African population and represents the first of its kind to be conducted in a South African population.

## Materials and methods

### Data available and recruitment

Data from 41 SCZ cases and 48 controls were available from the parent study, Shared Roots of Neuropsychiatric Disorders and Cardiovascular Disease Project (SR; HREC no. N13/08/115). The overarching aim of the parent study was to interrogate signatures (i.e., genomic, neural, cellular, and environmental) common to neuropsychiatric disorders and cardiovascular disease risk that could contribute to co-morbidity, symptom severity, and treatment outcomes.

Control participants were recruited through advertisements (i.e. print, radio and web), active recruitment within communities by a registered nurse, and word-of-mouth. Cases consisted of first-episode SCZ (FES total *n* = 24; recently diagnosed *n* = 8; within 5 years of diagnosis and treatment *n* = 16) and chronic SCZ cases (long-term and continuous presence of SCZ symptoms and/or treatment, *n* = 17). The FES cases were recruited from general and psychiatric hospitals and community health centres within the study catchment area following their first psychotic episode. The chronic schizophrenia cases were contacted and invited to participate in SR. Cases comprised individuals with a diagnosis of SCZ, schizophreniform, or schizoaffective disorder based on the Structured Clinical Interview for the Diagnostic and Statistical Manual of Mental Disorders, fourth edition (SCID-IV) [34], without intellectual disability. Symptoms and severity of SCZ were assessed using the Structured Clinical Interview for DSM-IV (SCID) and Positive and Negative Syndrome Scale (PANSS) [35].

The Mini International Neuropsychiatric Interview (MINI) version 6 [36] was used to exclude participants with other major psychiatric disorders or substance abuse. Additional exclusion criteria included antibiotic use four weeks prior to stool sampling, intellectual disability, severe physical illness, any neurological disorder, and FES cases treated with a long-acting depot antipsychotic medication. Childhood trauma was assessed using the Childhood Trauma Questionnaire (CTQ) [37] and total scores were calculated by adding scores from physical abuse, sexual abuse, emotional abuse, emotional neglect, and physical neglect. Participants with a total score of > 41 (representing the lowest level score indicative of neglect or abuse for each subscale included) were classified as having childhood trauma, as previously used by our research group [38].

The WHO STEPwise approach to Surveillance (STEPS) instrument [39] was used to examine body mass index (BMI). The medical history questionnaire determined smoking and alcohol habits. Participants were considered current smokers or current alcohol consumers if they smoked or used alcohol in the past 6 months. The harmonised Joint Interim Statement (JIS) criteria were used to assess metabolic syndrome (MetS) in participants [40].

### Sample collection and microbial DNA extraction

The collection of stool samples and subsequent DNA extraction was performed as previously described [41]. Briefly, stool samples were self-collected in pre-analytical sample processing (PSP) collection tubes (Stratec, Molecular, Birkenfeld, Germany) and stored at −20 °C in the Neuropsychiatric Genetics Laboratory (Department of Psychiatry, Faculty of Medicine and Health Sciences, Stellenbosch University, Cape Town) prior to microbial DNA extraction.

Microbial DNA extraction was done using the PSP Spin Stool DNA Plus kit (Stratec Molecular, Birkenfeld, Germany). For each batch of microbial DNA extraction performed, a negative (non-template buffer to control for large-scale cross-contamination) and positive (ZymoBIOMICS microbial mock community standards of known composition to assess the accuracy of results, Zymo Research, Cat # D6300) control was added to the microbial DNA extraction run. Quantity and quality of the microbial DNA were determined using an ultraviolet–visible (UV–Vis) Spectrometer Nano-Drop 2000 (Thermo Scientific, USA), and the Qubit 4 Fluorometer (Invitrogen, ThermoFisher Scientific, Massachusetts, USA). The fourth hypervariable region (V4) of the 16S rRNA gene was amplified using the following primer pair [42]:

515 F (5’ TCGTCGGCAGCGTCAGATGTGTATAAGAGACAGGTGYCAGCMGCCGCGGTAA)

806 R (5’ GTCTCGTGGGCTCGGAGATGTGTATAAGAGACAGGGACTACNVGGGTWTCTAAT).

The library preparation and sequencing were done at the Centre for Proteomic and Genomic Research (CPGR, Cape Town, South Africa). Illumina sequencing adapters and the dual-index barcodes were attached using the Nextera XT v2 Index Kit (Illumina Inc., San Diego, CA, USA). Sequencing libraries were pooled and diluted to 5 pM. The libraries were then sequenced with 250 bp pair-end reads on the Illumina MiSeq sequencing instrument using a MiSeq Reagent v2 Kit. The expected fragment size of the V4 amplicon is approximately 250–300 bp. Samples (depth = 60 000 reads) were converted to FASTQ files (forward, reverse and index) after sequencing using the BCL-to-FASTQ file converter bcl2fastq (ver. 2.17.1.14, Il-lumina, Inc.).

### Data preparation and taxa classification

The data preparation, quality control and taxa classification were done as previously described [41] in R Studio [43] using the *DADA2* pipeline (https://github.com/benjjneb/dada2) [44]. Quality control of the sequences consisted of assessing read quality profiles, filtering and trimming while maintaining overlap between the forward and reverse sequences. Error rates and inference of sample composition were estimated by a parametric error model, after which the sample inference algorithm was applied to the data to reduce redundancy and determine the number of unique sequences. Reads were merged with at least 12 bases in the overlap region [45]. After generating the amplicon sequencing variant (ASV) table and removing chimeras, a naïve Bayesian classifier method [44] was implemented to taxonomically classify the ASVs, using the Ribosomal Database Project (RDP) as a reference database [46]. The ASV table consisted of 3,628,011 reads, 12,204 taxa, and sparsity of 94.3%. Taxa observed in fewer than 15% of samples were eliminated from the ASV table as per a previous study [41].

### Statistical analysis of metadata

Statistical differences in metadata variables between cases and controls were assessed prior to diversity analysis. Categorical metadata included sex, MetS, childhood trauma, current alcohol consumption, and current smoker status. To assess the difference between cases and controls for categorical metadata variables, the Chi-square (χ2) test was used. The results were expressed in the form of numbers (N) and percentages (%). According to Shapiro–Wilk’s normality test, age and BMI were not normally distributed. Therefore, Mann–Whitney U-test (U test)[47] was used to assess differences between cases and controls with the results expressed as medians and interquartile ranges (IQR). Significance was defined as *p* < 0.05.

### Diversity data analysis

Alpha- and beta-diversity analyses were conducted using the *QIIME2 q2-diversity* plugin [48], and differential taxa abundance testing was done using Analysis of Compositions of Microbiomes with Bias Correction (ANCOM-BC) [49] in QIIME2, using the *q2-composition* plugin [48]. *P*-values were adjusted for multiple testing according to Benjamini-Hochberg’s procedure [50] and represented as *q*-values. The statistical significance level was set at α = 0.05 for all tests.

#### Alpha-diversity analysis

Alpha-diversity measures were calculated to assess the microbial diversity within individual samples in groups. The Shannon and Simpson diversity estimators [51] were used to estimate species richness and evenness, with Shannon being more sensitive to species richness whereas Simpson is more sensitive to species evenness [52].

#### Covariate selection for beta-diversity

The Multivariate Association with Linear Models 2 (MaAsLin2) package [53] in R Studio [43] was used to determine the association between metadata variables and microbial community abundance. All the potential covariates were assessed through the multivariate *MaAslin2* function using the case–control status (“Control”) as a reference. Metadata variables were included as covariates in subsequent analyses of beta-diversity if a statistically significant result for MaAsLin2 was observed (*q* < 0.05).

#### Principal Coordinates Analysis (PCoA)

A principal coordinates analysis (PCoA) was used to assess ordination and visualize the variance and dissimilarity of taxa composition between samples based on Euclidean distances [54]. The first three principal coordinates were utilized, as they represent the largest eigenvalues for a three-dimensional PCoA plot [54]. The ordination plot was used to examine any potential clusters based on case–control status and covariates.

#### Permutational multivariate analysis of variance (PERMANOVA) tests

The permutational multivariate analysis of variance (PERMANOVA) adonis test [55] was performed to determine if there were differences between microbial community samples (permutations = 999; *α* = 0.05). Pairwise comparisons between cases and controls were conducted to determine specific associations.

#### Differential abundance analysis

Taxa associated with case–control status was assessed with ANCOM-BC [49], using control status as the reference. Microbial taxa were considered significantly differentially abundant at *q* < 0.05. The associated log-fold change was calculated during the ANCOM-BC diversity analysis [49].

## Results

### Cohort description

There were no statistically significant differences between the SCZ case and control groups for n sex (*p* = 0.093), age (*p* = 0.332), BMI (*p* = 0.283), MetS (*p* = 0.061), childhood trauma (*p* = 0.123), alcohol consumption (*p* = 0.083), or smoking (*p* = 0.253), as shown in Table 1. SCZ cases were all on psychotropic medication, comprising different medication combinations and dosages. Five control participants reported current use of medication for depression (*n* = 2), anxiety (*n* = 2) and appetite suppression (*n* = 1).

**Table 1:** Clinical and demographic characteristics of cohort

Variables	Cases (*n* = 41)	Controls (*n* = 48)	*p*-value
Sex (female n, %)	14 (34.2)	26 (54.2)	0.093 ^χ^
Age (years)(median, IQR)	27.50 (10.46)	27.33 (9.48)	0.332 ^U^
BMI (median, IQR)	23.97 (9.89)	23.765 (7.29)	0.283 ^U^
MetS (yes n, %)	11 (26.8)	5 (10.4)	0.061 ^χ^
Childhood trauma (yes n, %)	28 (68.3)	26 (54.2)	0.123 ^χ^
Current alcohol consumption (yes n, %)	14 (34.2)	27 (56.3)	0.083 ^χ^
Current smoker (yes n, %)	23 (56.1)	18 (37.5)	0.253 ^χ^

*BMI* body mass index, *MetS* metabolic syndrome, *n* number, *IQR* interquartile range (Q3 – Q1)

^χ^ Chi-square; ^U^ Mann–Whitney U test

* *p* < 0.05

Most cases (*n* = 24, 58.5%) had FES (recently diagnosed *n* = 8; within 5 years of diagnosis and treatment *n* = 16). The remaining cases (*n* = 17, 41.5%) had chronic SCZ. These subgroups were analysed together to improve statistical power. Additionally, a combined analysis could improve the generalizability of the results, making it more applicable to the broader SCZ population.

### Alpha-diversity analysis

No significant association was found between alpha-diversity measures and case–control status (Shannon *q* = 0.090; Simpson *q* = 0.174).

### Beta-diversity analysis

#### Covariates for beta-diversity analysis

*MaAsLin2* assessed the association between metadata variables (sex, age, BMI, MetS, childhood trauma, current alcohol consumption, and current smoking status) and the relative abundance of taxa in cases and controls. Metadata variables with a statistically significant microbial abundance differences included sex (*Subdoligranulum*, *q* = 0.041), current alcohol consumption status (*Dehalobacterium*, *q* = 0.041; Enterobacteriaceae, *q* = 0.037) and current smoking status (*SMB53*; *q* = 0.041). Therefore sex, current alcohol consumption status, and current smoking status were included as covariates when investigating beta-diversity.

#### Permutational multivariate analysis of variance analysis of beta-diversity

Gut microbial communities were not significantly different between cases and controls (*p* = 0.547) after correcting for the contribution of sex, current smoking status, and current alcohol consumption (Table 2). Current smoking status significantly contributed to variance between samples in the cohort (*p* = 0.036) and was subsequently included in the PCoA plot.

**Table 2:** Results of the adonis test

Model	R^2^	*P*-value
Status	0.011	0.547
Sex	0.013	0.063
Current alcohol consumption	0.013	0.090
Current smoker	0.015	0.036*
Residuals	0.948	NA
Total	1.000	NA

*Status: case or control*

* *p* < 0.05

#### Principal coordinates analysis

**Fig. 1:**
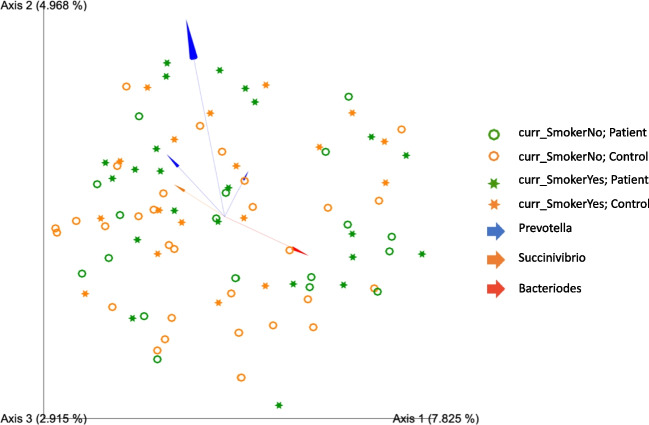
Principal coordinates analysis plot illustrating genus-level gut microbial community and composition analysis for SCZ cases and controls. PCoA was used to assess the community variance (symbols) and compositional variance (vectors) of SCZ cases and controls. The distance between the symbols approximates the dissimilarities between the microbial communities of samples (q = 0.124), while the vectors indicate the taxonomic variance driven by *Bacteroides, Prevotella,* and *Succinivibrio*. Axis 1 explained 7.825% of the variance, while axes 2 and 3 explained 4.968% and 2.915%, respectively

Current smoking status was included in the PCoA plot as it significantly contributed to variance between samples (*p* = 0.036). The PCoA showed no statistically significant difference in the variance or dissimilarity of the microbial composition between case and control participants (*q* = 0.124). In Fig. 1, the distance between the symbols represents an approximation of the dissimilarity between samples. Axis 1 explained 7.825% of the variance, while axes 2 and 3 explained 4.968% and 2.915%, respectively. Gut microbial composition variance was primarily driven by *Bacteroides, Prevotella,* and *Succinivibrio* as shown by the red, blue, and orange arrows, respectively (Fig. 1).

### Differential abundance of taxa associated with schizophrenia

**Fig. 2:**
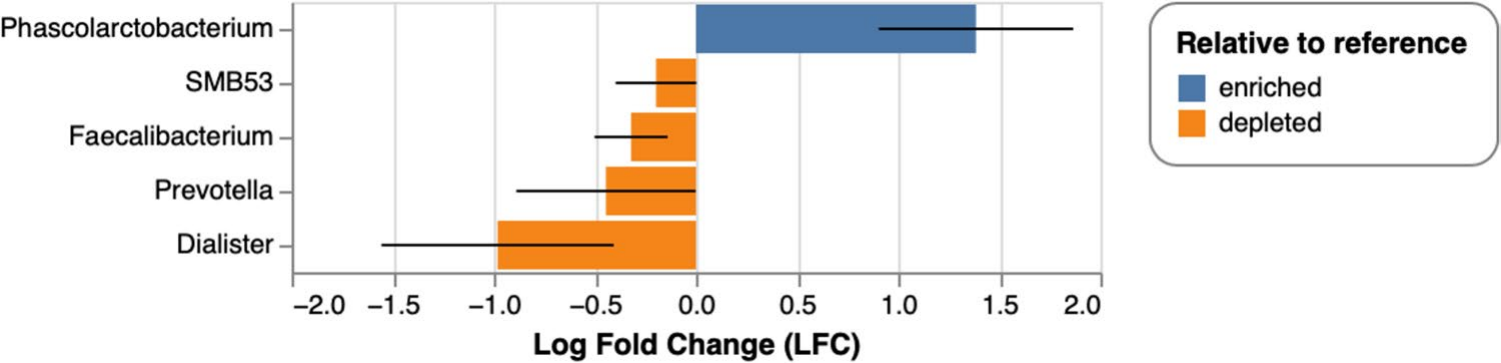
Statistically significant differences in the relative abundance of taxa between SCZ cases and controls, prior to correction for multiple testing. The relative abundance of taxa that were statistically different in SCZ cases compared to controls was assessed using ANCOM-BC. Enriched or depleted relative abundances in SCZ cases versus controls were identified based on the log-fold change and considered statistically significant at a threshold of *p* = 0.05. Significantly decreased relative abundances of genera *Prevotella* (*p* = 0.037; *q* = 0.778), *Faecalibacterium* (*p* = 0.032; *q* = 0.778), *Dialister* (*p* = 0.043; *q* = 0.778), and *SMB53* (*p* = 0.012; *q* = 0.513) were associated with SCZ, whilst the relative abundance of the genus *Phascolarctobacterium* was enriched in SCZ cases compared to controls (*p* = 0.002; *q* = 0.279)

The relative abundances of *Prevotella* (*p* = 0.037; *q* = 0.778)*, Faecalibacterium* (*p* = 0.032; *q* = 0.778)*, Phascolarctobacterium* (*p* = 0.002; *q* = 0.279)*, Dialister* (*p* = 0.043; *q* = 0.778)*,* and *SMB53* (*p* = 0.012; *q* = 0.513) were associated with SCZ case–control status. However, these associations did not withstand correction for multiple testing. All relative taxa abundances, except for *Phascolarctobacterium,* were depleted in SCZ cases compared to controls (Fig. 2).

## Discussion

In the current study, we investigated potential associations between microbial taxa and SCZ in a South African cohort. We found no statistically significant differences in alpha- or beta-diversity between cases and controls. However, we did observe individual differences in relative taxa abundance between SCZ cases and controls, including the reduced relative abundances of *Prevotella, Faecalibacterium, Dialister* and *SMB53,* and the enriched relative abundance of *Phascolarctobacterium* in SCZ cases versus controls. However, these associations did not withstand correcting for multiple testing.

Previous reports on alpha-diversity in SCZ investigations are inconsistent [24], with observations of both decreased [28] and increased alpha-diversity [23] in SCZ cases versus controls. The majority of studies, however, report no difference in alpha-diversity [4, 22, 25, 27, 29, 56], in line with our observation. Alpha-diversity has previously been associated with external factors, such as medication or environment (e.g. diet and geography), which may contribute to these discrepancies in results among studies [24, 57]. Differences in beta-diversity in SCZ cohorts are more consistently reported by previous studies [24], which is in contrast to our observation of no statistically significant difference in beta-diversity. The discrepancies between our results with other studies may be due to sample size limiting statistical power to detect differences between cases and controls. Additionally, discrepancies could be due to environmental factors, cohort characteristics or covariates included in the analysis ta.

In unadjusted analysis, relative abundance differences in individual taxa between cases and controls align with previous studies that have reported an association between *Faecalibacterium* [22, 28, 57–59] and *Dialister* [23, 60] and SCZ. Our observation of reduced relative abundance of *Prevotella* in SCZ cases contrasts with findings from some previous studies [22, 28, 60–62]. However, our results should be interpreted with caution as our observations did not survive correction for multiple testing. Differences in the relative abundances of *Prevotella*, *Phascolarctobacterium*, *Dialister* and *Faecalibacterium* were found to be associated with several neurological and psychiatric disorders in a recent study [63], suggesting that these taxa may be involved in disease aetiology. Potential mechanisms behind the associations of *Prevotella, Faecalibacterium*, and *Dialister* with SCZ can be speculated based on SCFA produced by these taxa [57]. *Prevotella* is an acetate-producing taxon*,* whereas *Faecalibacterium* is a butyrate-producing taxon, and *Dialister* produces propionate [64]. Alteration in their relative abundance may affect the availability of SCFA produced by these taxa. A decrease in SCFA availability, for example, has been suggested to be associated with increased risk for SCZ [16], considering that SCFA are known to play in gut barrier maintenance, immune response, and brain function [58, 65, 66]. Decrease in SCFA levels, particularly acetate, butyrate, and propionate, is associated with inflammation and neuroinflammation [58, 65, 66], which has been implicated in SCZ [10, 23, 67]. Butyrate has been shown to have anti-inflammatory properties [58, 66], therefore, a decreased relative abundance of butyrate-producing taxa, such as *Faecalibacterium* [11], may lead to higher levels of low-grade systemic inflammation. Furthermore, acetate and propionate are known to have neuroprotective and anti-neuroinflammatory effects [65, 68, 69], potentially affected by decreased SCFA levels. This suggests that there may be an association of these genera in inflammation due to the changes in SCFA levels in SCZ [4, 15, 70]. However, these hypotheses need to be tested as SCFA concentrations were not assessed in the current study. Furthermore, various species and strains within a single genus may produce varying concentrations of the same SCFA, or different SCFA altogether.

This is the first study investigating the gut microbiome and SCZ in South Africa. As previous studies have primarily been conducted in Asian, European, or American cohorts [24, 58], direct comparison is difficult as geographic location has been associated with gut microbial composition [24]. Discrepancies in findings across studies may also be influenced by sample size, the lack of standardised pipelines of gut microbial analysis, specific population characteristics, and environmental factors such as lifestyle and medications [9], as the gut microbiome is highly dynamic and malleable [25]. To gain further insights into gut microbial profiles in SCZ, future studies will require larger samples, uniform study designs, consistent methodologies, and harmonisation of covariates known to affect the gut microbiome (e.g., stool consistency, medication, and diet) [71].

This study contributes to the current body of knowledge by providing preliminary insights into the potential associations between the gut microbiome and SCZ in a South African population. Although the results were not statistically significant after correction for multiple testing, they hint at possible associations that align with reports from previous studies and lay the groundwork for future functional investigation of the gut microbiome and SCZ to improve our understanding of SCZ pathogenesis.

## Limitations

The main limitation is the small sample size and subsequent lack of statistical power (power = 0.643, effect size (d) = 0.5, *⍺* = 0.05) that could have contributed to Type II error (false negative results), necessitating replication in a larger independent sample [72, 73]. Moreover, this study only assessed the relative abundance of taxa at the genus level as 16S rRNA gene sequencing lacks accurate species-level data. Future studies should therefore consider the use of shotgun metagenomic sequencing. The cross-sectional design limits our ability to infer causation. Additionally, there are many factors, such as diet and medication, which may affect the gut microbial composition but were not included in the current study. We repeated the analysis after excluding the five control participants who reported current use of medication for depression (*n* = 2), anxiety (*n* = 2) and appetite suppression (*n* = 1). However, this did not affect the statistical significance of our findings. The effect of this medication use may have been too small to be detected. Furthermore, it would be important to stratify by FES and chronic SCZ cases, but the sample size did not allow this. Lastly, the SCZ patient sample was recruited from a localised region of the country and is not ethnically and culturally representative of all SCZ patients in SA. Larger studies that are more geographically representative are needed to validate and strengthen these findings.

## Conclusion

Results suggest potential associations between SCFA-producing genera and SCZ that warrant further investigation. Alterations in the gut microbiome composition in SCZ could lead to changes in SCFA production, potentially contributing to the pathophysiology of SCZ via the MGB axis. Longitudinal studies in larger samples may advance our understanding of the MGB axis and its relevance to SCZ pathogenesis.

## Data Availability

The datasets presented in this article are not readily available due to ethical and legal restrictions. Requests to access the datasets should be directed to SMJH (smjh@sun.ac.za). The authors are open to collaborating and sharing data within the limits of ethical review restrictions and data transfer policies of Stellenbosch University.

## Abbreviations

adonis: Adonis permutation-based statistical test
ANCOM-BC: Analysis of Compositions of Microbiomes with Bias Correction
BBB: Blood–brain barrier
BMI: Body mass index
CNS: Central nervous system
LPS: Lipopolysaccharides
MaAsLin2: Multivariate Association with Linear Models 2
MetS: Metabolic syndrome
MGB: Microbiome-gut-brain
MINI: MINI International Neuropsychiatric Interview
PANSS: Positive and Negative Syndrome Scale
PCoA: Principal coordinates analysis
PERMANOVA: Permutational multivariate analysis of variance
PSP: Pre-analytical sample processing
RDP: Ribosomal Database Project
SCFA: Short-chain fatty acid
SCZ: Schizophrenia
SR: Understanding the SHARED ROOTS of Neuropsychiatric Disorders and Modifiable Risk Factors for Cardiovascular Disease’ or SHARED ROOTS
STEPS: WHO STEPwise approach to Surveillance
χ2: Chi-square
